# Microscopic origins of performance losses in highly efficient Cu(In,Ga)Se_2_ thin-film solar cells

**DOI:** 10.1038/s41467-020-17507-8

**Published:** 2020-08-21

**Authors:** Maximilian Krause, Aleksandra Nikolaeva, Matthias Maiberg, Philip Jackson, Dimitrios Hariskos, Wolfram Witte, José A. Márquez, Sergej Levcenko, Thomas Unold, Roland Scheer, Daniel Abou-Ras

**Affiliations:** 1 Helmholtz-Zentrum Berlin, Hahn-Meitner-Platz 1, 14109 Berlin, Germany; 2 Institute of Physics, Martin-Luther University Halle-Wittenberg, Von-Danckelmann-Platz 3, 06120 Halle, Germany; 3 Zentrum für Sonnenenergie- und Wasserstoff-Forschung Baden-Württemberg (ZSW), Meitnerstr. 1, 70563 Stuttgart, Germany

**Keywords:** Solar cells, Solar cells, Characterization and analytical techniques

## Abstract

Thin-film solar cells based on polycrystalline absorbers have reached very high conversion efficiencies of up to 23-25%. In order to elucidate the limiting factors that need to be overcome for even higher efficiency levels, it is essential to investigate microscopic origins of loss mechanisms in these devices. In the present work, a high efficiency (21% without anti-reflection coating) copper indium gallium diselenide (CIGSe) solar cell is characterized by means of a correlative microscopy approach and corroborated by means of photoluminescence spectroscopy. The values obtained by the experimental characterization are used as input parameters for two-dimensional device simulations, for which a real microstructure was used. It can be shown that electrostatic potential and lifetime fluctuations exhibit no substantial impact on the device performance. In contrast, nonradiative recombination at random grain boundaries can be identified as a significant loss mechanism for CIGSe solar cells, even for devices at a very high performance level.

## Abstract (web summary)

Achieving higher efficiencies for thin-film solar cells always requires identification of the limiting factors. Here Krause et al. show that inhomogeneously distributed net doping or lifetime have little impact while recombination at grain boundaries is one of the main loss mechanisms for high performance Cu(In,Ga)Se_2_ solar cells.

## Introduction

Thin-film solar cells with polycrystalline absorber layers exhibit high power-conversion efficiencies of up to 23–25%^1–3^. In spite of their excellent photovoltaic (PV) performance, it is apparent, when comparing the PV parameters of record devices with the theoretical radiative limit^4^, that various loss mechanisms are present in the solar cell devices. For the present work, we chose Cu(In,Ga)Se_2_ (CIGSe) solar cells, for which the open-circuit voltage (*V*_oc_) and the fill factor (FF) are limited by nonradiative recombination, as an exemplary case, in order to demonstrate the importance of microscopic and correlative analysis, combining experimental and simulation methods. We were able to provide answers to the question where nonradiative recombination processes occur dominantly on the microscopic scale and which possible recombination paths are irrelevant for CIGSe solar cells.

In order to gain access to this information, a CIGSe solar cell, which exhibits a high conversion efficiency of about 21% without anti-reflection coating (ARC), was characterized intensely in the present work. We have employed not only various scanning electron microscopy (SEM) techniques as well as photoluminescence (PL) and capacitance–voltage (CV) analysis but have also performed two-dimensional device simulations in order to corroborate the experimental results.

We show that enhanced recombination at grain boundaries (GBs) can be identified as one origin for the limitation of the *V*_oc_, and that electrostatic potential as well as electron-lifetime fluctuations within the CIGSe layer play negligible roles in terms of solar cell efficiency.

## Results

### Solar cell device performance

The theoretical device performance at the radiative limit was calculated using the approach described in ref. ^5^ using an AM1.5 solar spectrum, an absolute temperature of 300 K, and a band-gap energy of 1.11 eV, as determined by external quantum efficiency (EQE) measurements. The corresponding theoretical PV parameters are compared in Table 1 with the values measured on the solar cell. It is apparent that the measured *V*_oc_, short-circuit current density (*j*_sc_), and FF contribute similarly to the loss in conversion efficiency when compared with the theoretical (radiative) limit.

**Table 1:** Photovoltaic parameters from experiment and simulations.

	*V*_oc_ (mV)	*j*_sc_ (mA cm^−2^)	FF (%)	*η* (%)
Experimental	720	36.6	79	20.8
Theoretical	867	44.1	87	33.2
Simulation	720 (750)	36.7	80 (82)	21.1 (22.4)

Solar cell parameters (open-circuit voltage *V*_oc_, short-circuit current density *j*_sc_, fill factor FF, and the power conversion efficiency *η*) of the investigated CIGSe solar cell (total area; without anti-reflective coating) as well as the theoretical values corresponding to a band-gap energy of 1.11 eV and an AM1.5 G power spectrum calculated by the Shockley–Queisser approach^5^. In addition, simulated photovoltaic parameters are listed. The simulation was performed based on the parameter set in Supplementary Table 3. The *V*_oc_ value in parentheses (720 mV) is the one for a CIGSe layer exhibiting band-gap fluctuations. The FF value for this *V*_oc_ becomes 80% instead of 82% (using the formalism given by ref. ^34^, with a diode-ideality factor of 1.4 and a series resistance of 0.5 Ω cm^2^ (determined from the *j*–*V* data of the investigated solar cell), leading to a decreased conversion efficiency of 21.1%.

### Absorber morphology characterization

**Fig. 1:**
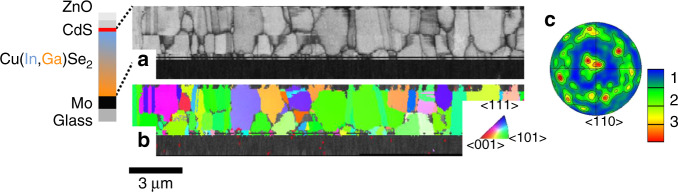
Electron backscatter diffraction maps. **a** Cross-sectional EBSD pattern-quality map of the CIGSe in the analyzed solar cell stack and **b** orientation distribution map with local orientations perpendicular to the substrate highlighted by false colors within the related inverse pole figure, as well as **c** the pole figure for the <110> orientation (with the multiples of random values given by false colors).

The microstructure of the CIGSe thin films was analyzed thoroughly by means of electron backscatter diffraction (EBSD) (Fig. 1). The average grain size of this layer was about 0.5 µm (average diameter of circles that have the same area as the grains). Evaluation of the local orientations of the grains revealed a weak, preferred orientation in the <110> crystal direction perpendicular to the substrate, at a low multiples-over-random-distribution value of about 2–4 (see pole figure in Fig. 1c). As already shown by Contreras et al.^6^ as well as by Abou-Ras et al.^7^, the preferred orientation of <110> versus <111> does not have a direct impact on the device performance; rather, the film texture can be considered a signature of the Se/metal flux ratio during the CIGSe coevaporation and thus of the concentration of Se vacancies in the produced CIGSe thin film. Although a large number of GBs exhibit an orientation perpendicular to the substrate, also a substantial fraction is oriented parallel to the substrate, i.e., possibly impeding the current flow of the generated charge carriers via enhanced nonradiative recombination.

### Elemental analysis by energy-dispersive X-ray spectrometry (EDX)

**Fig. 2:**
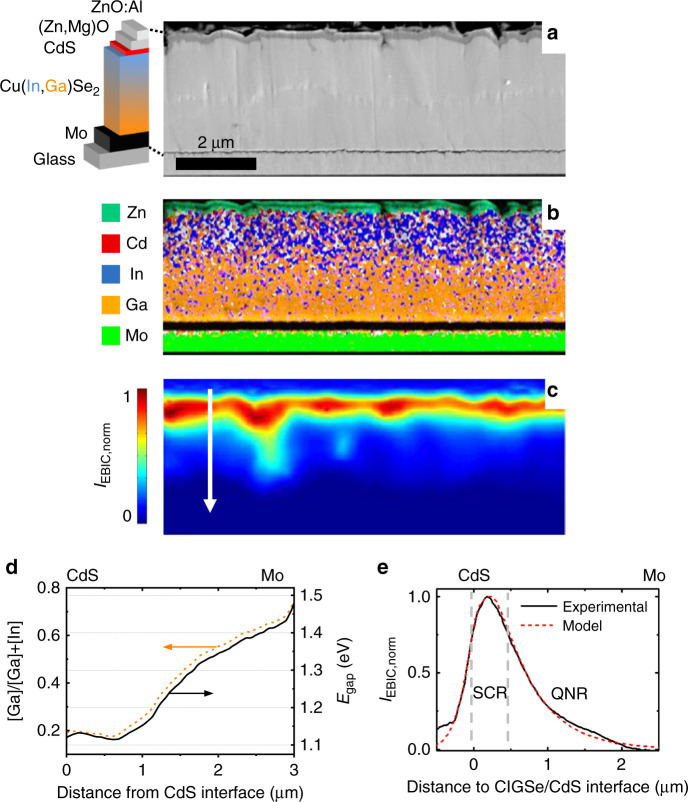
Electron beam-induced current analysis and energy-dispersive X-ray spectroscopy. **a** Cross-sectional SEM image, **b** a corresponding EDX elemental distribution map, and **c** EBIC image (in which the EBIC values are given by false colors), acquired at the identical position of a ZnO:Al/(Zn,Mg)O/CdS/CIGSe/Mo/glass stack. The EDX map is composed of signals from Ga-L (orange) and In-L X-ray lines (blue) in the CIGSe absorber layer as well as from Zn-L lines (turquoise) in the window layer and also Mo-L lines (green) in the Mo back contact. A weak signal from Cd-L lines (red) is also visible in the buffer layer. **d** [Ga]/([Ga]+[In]) ratio and corresponding calculated band-gap shift (according to ref. ^45^). **e** Extracted EBIC profile along the white arrow in **c** for a beam energy of 12 keV (black solid line) and analytic model (red dashed line). The space-charge (SCR) and quasi-neutral regions (QNR) are highlighted by vertical dashed lines.

The elemental distributions across the ZnO:Al/(Zn,Mg)O/CdS/CIGSe/Mo/glass stack were analyzed by means of EDX. The corresponding elemental distribution maps are given in Fig. 2a, b. From the [Ga]/([Ga]+[In]) ratio and the corresponding band-gap energy (Fig. 2d), it can be seen that the CIGSe absorber exhibits a double gradient, with decreasing [Ga] from the Mo back contact, shallow increase toward the CdS buffer layer, and a local band-gap energy minimum of about 1.11 eV (notch) at about 0.6 µm away from the CIGSe/CdS interface.

### Charge carrier collection behavior

The collection behavior in the CIGSe solar cell was investigated by means of electron-beam-induced current (EBIC) analyses (Fig. 2c). From the EBIC images acquired at various beam energies of 6–15 keV, linescans were extracted (exemplarily shown in Fig. 2e) and evaluated using an analytical approach described in refs. ^8,9^, in order to obtain values for the widths of the space-charge regions (SCRs). The acquisition and evaluation of EBIC signals at various beam energies (Supplementary Fig. 2) also allowed for an assessment of the influence of surface recombination. Within the following sections, various types of inhomogeneities were investigated. More specifically, we will elucidate the influences of electrostatic potential, band-gap, as well as electron-lifetime fluctuations on the device performance of CIGSe solar cells. Since we will show in the sections “Cathodoluminescence (CL) behavior of the CIGSe absorber” and “Two-dimensional device simulations” that the band-gap and lifetime fluctuations are negligible, we will concentrate in the present section only on electrostatic potential fluctuations. Their magnitude can be estimated by evaluating the lateral variations in the widths of the SCR.

From Fig. 2c and Supplementary Fig. 2, it is apparent that lateral fluctuations are present in the measured widths of the SCRs, which indicate inhomogeneous electrostatic potential and lifetime distributions in the CIGSe absorber layer as well as at the interface between CIGSe and the CdS buffer. The electrostatic potential fluctuations can be caused by variations in net-doping densities present in the *p*-type CIGSe absorber (*N*_A_) as well as in the *n*-type ZnO:Al/(Zn,Mg)O/CdS stack (*N*_D_) or by charge densities *N*_IF_ present at the interface between CIGSe and CBD-CdS. In the following subsections, the fluctuations in the widths of the SCRs will be evaluated as function of lateral variations in *N*_A_, *N*_D_, and *N*_IF_.

By evaluating linescans extracted perpendicular to the substrate from the EBIC images (at varying beam energy) in Supplementary Fig. 2, the magnitudes for the average value and for the fluctuations of the width of the SCR were determined to be about (230 ± 60) nm. We note that these values were calculated as average and standard deviation from ten individual values determined for the extracted EBIC linescans and that this value did not vary substantially for the various beam energies applied (see Supplementary Fig. 2).

Considering a *p*–*n* heterojunction with *p*-type absorber and *n*-type buffer/window layer, the width of the SCR on the absorber can be expressed generally as “Equation (2.27) in ref. ^10^”

1 $$w_{\mathrm{a}}\left( V \right) = z\varepsilon _{\mathrm{a}}N_{{\mathrm{IF}}}/{\it{\Omega }} + ( {\varepsilon _{\mathrm{a}}\varepsilon _{\mathrm{w}}N_{{\mathrm{D,w}}}\left[ {2{\it{\Omega }}/e\left( {V_{{\mathrm{bi}}} - V} \right)-N_{{\mathrm{IF}}}^2} \right]/N_{{\mathrm{A,a}}}{\it{\Omega }}^2} )^{1/2},$$

where *z* gives the sign of the interface charge density *N*_IF_, *ε*_a_ and *ε*_w_ are the dielectric permittivities of absorber and window layer, respectively, Ω = *ε*_w_*N*_D,w_ + *ε*_a_*N*_A,a_, *N*_D,w_ and *N*_A,a_ are the net-doping densities of the window and absorber, respectively, and *V*_bi_ is the built-in potential. Moreover, at a heterojunction, *V*_bi_ does not only depend on *N*_D,w_ but also on the offsets Δ*E*_C_ and Δ*E*_V_ in the conduction and valence bands, respectively^11^. We keep in mind that the fluctuations in *w*_a_ can be affected by lateral variations in *N*_IF_. However, Eq. (1) contains a complex equation with a large number of unknown quantities.

Therefore, in order to obtain a simple expression for a rough estimate of the doping concentration in the absorber, we assume *N*_IF_ = 0 and set *V* = 0 and *N*_D,w_ > > *N*_A,a_ (as in CIGSe solar cells), i.e., the relationship between *w*_a_ and *N*_A,a_ at a homojunction is,

2 $$w_{\mathrm{a}} = ( {2\varepsilon _{\mathrm{a}}V_{{\mathrm{bi}}}/eN_{{\mathrm{A,a}}}} )^{1/2},$$

with^11^

3 $$V_{{\mathrm{bi}}} = k_{\mathrm{B}}T/e\,{\mathrm{ln}}( {N_{{\mathrm{A,a}}}N_{{\mathrm{D,w}}}/n_{\mathrm{i}}^2} ).$$

Values for *N*_D,w_ were assumed to be 1 × 10^18^ cm^−3^ as well as *n*_i_ (the intrinsic charge-carrier density) to be 5 × 10^9^ cm^−3^ according to refs. ^12,13^, and *ε*_a_ were set to 13.6^14^. Using Eqs. (2) and (3), we determined *N*_A,a_ to about (2 ± 1) × 10^16^ cm^−3^ (average value and lateral fluctuation). This value agrees very well with the value obtained from the minimum in the CV profiling data shown in Supplementary Fig. 1.

Finally, a rough estimation for the impact on the device performance of inhomogeneities concerning the electrostatic potential can be obtained using the analytic model of Rau and Werner^13,15^. We will consider only electrostatic potential fluctuations in the following.

The amplitude of the electrostatic potential fluctuation *φ* is given by^11^

4 $$\varphi = k_{\mathrm{B}}T/e\,{\mathrm{ln}}( {N_{{\mathrm{A}},1}/N_{{\mathrm{A}},2}} ),$$

where *k*_B_*T* is the thermal energy, *e* the elementary charge, and *N*_A,1_ and *N*_A,2_ are the net-doping densities at two neighboring positions in the CIGSe absorber. Assuming that the standard deviation (i.e., lateral fluctuation) of *φ* is given by *σ*, a relation between the *V*_oc_ loss, *V*_oc,loss_, and *σ* can be described by^15^

5 $$V_{{\mathrm{oc,loss}}} = \sigma ^2/2ek_{\mathrm{B}}T.$$

When inserting varying net-doping values *N*_A,1_ and *N*_A,2_ obtained by EBIC into Eq. (4), we obtained a standard deviation of the fluctuation amplitudes of about 30 meV. Corresponding losses in *V*_oc_ can be estimated via Eq. (5) to about 15–20 mV, which is a rather small value as compared with a *V*_oc_ deficit of around 120 mV.

In addition to the width of the SCR, also the signal decays in the quasi-neutral region (QNR) were evaluated from the extracted EBIC profiles (Fig. 2e), in order to obtain values for the effective electron diffusion lengths. These values were on the order of several 100 nm. It can be expected that the real values present in the CIGSe bulk are substantially larger, since at the cross-section surface, enhanced nonradiative recombination takes places owing to increased defect densities. The real electron diffusion lengths in the QNRs of the investigated CIGSe absorbers can be estimated roughly to about 2–3 µm for assumed surface-recombination velocities of 10^5^–10^6^ cm s^−1^ using the approach described in ref. ^16^. However, we note that this approach is not very accurate, and a much more reliable way to obtain values for electron diffusion lengths is to perform absolute PL measurements, as described further below.

Finally, we extracted EBIC linescans also across GBs in order to determine the recombination velocities at these planar defects^8^. However, we found that the signal-to-noise ratios of various EBIC measurements were too poor for an unambiguous determination of recombination velocities at the GBs. Nevertheless, for the very high-efficient CIGSe solar cells with rubidium fluoride post-deposition treatment (RbF PDT) analyzed for the present work, no enhanced EBIC signals as compared with signal levels in the grain interiors were detected at any GB. Our observation is in contrast to other reports in the literature by Raghuwanshi et al.^17^. These authors found enhanced carrier collection at >50% of the investigated GBs as compared with the grain interiors on a CIGSe sample that was not subjected to alkali-metal PDT. Thus we cannot confirm enhanced collection of charge carriers at GBs in the highly efficient CIGSe solar cell with RbF PDT.

### Cathodoluminescence (CL) behavior of the CIGSe absorber

**Fig. 3:**
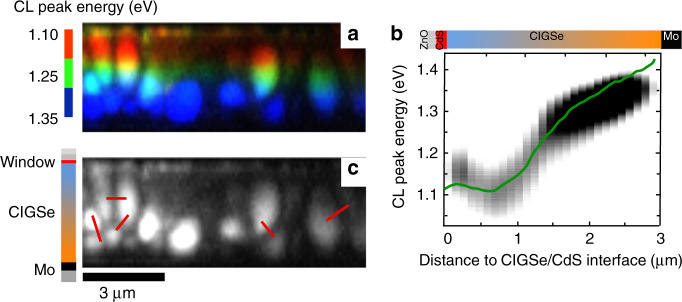
Cathodoluminescence results. **a** Hyperspectral map acquired at 10 keV representing the peak energies in the CL spectra by false colors, which can be (roughly) assigned to the band-gap energies at the corresponding positions in the map. **b** Linescan extracted from the hyperspectral CL map, showing presumably the distribution of the CL peak energies perpendicular to the substrate, which agrees well with the one expected from the Ga distribution (green, given in Fig. 2d). **c** CL intensity map, where the red lines mark the positions at which profiles were extracted across grain boundaries.

A hyperspectral CL map acquired at room temperature and at an acceleration voltage of 10 keV on the identical cross-sectional specimen as the previous results is shown in Fig. 3a, representing the raw data of the CL emission peak energies in the spectra as false colors (in each pixel, an entire spectrum was recorded). In order to get an estimate of the lateral distribution of band-gap energies, one can assume that these CL emission peak energies reflect the local band-gap energies. The corresponding values are found to run from about 1.15 eV (close to the CdS layer) to about 1.35 eV (near the Mo back contact), with a local minimum at about 1.1 eV. A corresponding linescan perpendicular to the substrate depicts the CL peak energy distribution in Fig. 3b. For comparison, the band-gap distribution calculated from the Ga distribution (Fig. 2d) is superimposed on the distribution of CL peak energies.

Figure 3c gives the CL intensity distribution across the identical area as in Fig. 3a. It is apparent that there are fluctuations in intensity not only between neighboring grains but also within individual grains. These fluctuations may be attributed to variations in net-doping density^18,19^ and/or minority carrier lifetime in the CIGSe absorber layer. At some positions, the CL intensity is much lower than at others; we cannot exclude influences on the CL intensity by the surface quality after the polishing procedure of the cross-section preparation, which may affect considerably nonradiative recombination at the cross-section surface.

**Fig. 4:**
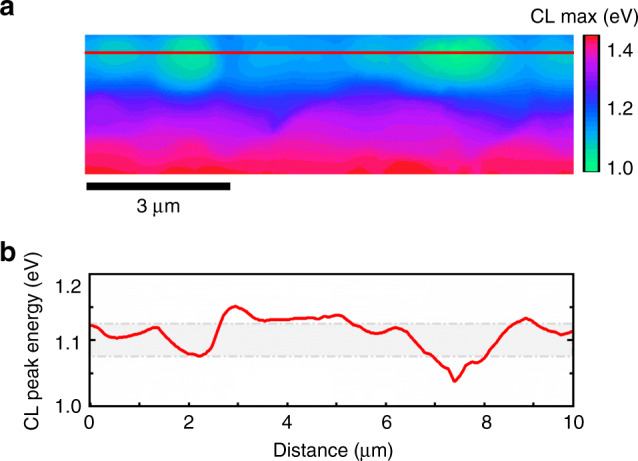
Evaluation of cathodoluminescence peak energy. **a** CL peak energy distribution map, from the identical cross-section area as in Fig. 3, and **b** extracted profile (red line in **a**) reveals band-gap energy fluctuations with a standard deviation (gray area) of <25 meV near the interface to the CdS buffer layer.

From the CL data displayed in Fig. 3a, we wanted to estimate the extent of band-gap fluctuations in the CIGSe layer. Therefore, we evaluated the lateral distribution of the emission peaks corresponding to band–band transitions in the individual CL spectra. In order to facilitate this evaluation, the luminescence intensities in each pixel were normalized, and the corresponding CL peaks were modeled by Gaussian functions, which results in the peak energy distribution map given in Fig. 4a. Even at positions with the largest lateral variations in peak energy, the standard deviations of the band-gap fluctuations did not exceed 25 meV (Fig. 4b). We should add that these fluctuations were investigated by means of CL maps that exhibit spatial resolutions of about 50–100 nm. This is roughly the lower limit down to which the band-gap fluctuations can be detected.

A rough estimation for the impact on the device performance by these band-gap fluctuations can be performed using again the analytic model of Rau and Werner^13,15^. A relationship between *V*_oc_ and the standard deviation of the fluctuation amplitude *σ* is described by Eq. (5). For *σ* *=* 25 meV, losses in *V*_oc_ amount to about 10 mV, which is (also again) a rather small value compared with the actual *V*_oc_ deficit.

**Fig. 5:**
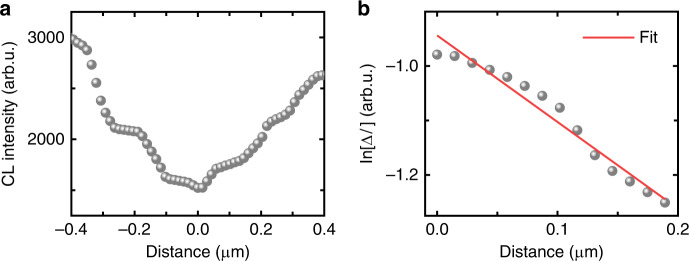
Modeling of grain boundary recombination velocity. Extracted CL profiles across a GB (**a**), GB is located at zero position. Corresponding ln[ΔI] plot for positive distance values with linear regression (**b**). The corresponding equation for the linear regression curve is ln[Δ*I*(*x*)] = −*x*/0.42 + ln(0.73/(0.73 + 1)) where *x* is the distance.

At random GBs, nonradiative recombination is enhanced and thus CL emission is reduced. In contrast, most twin boundaries in CIGSe thin films do not reveal significant reduction in CL signals^8,20,21^. From the CL intensity map (Fig. 3c), extracted profiles across random GBs (red lines in Fig. 3c) are presented in Fig. 5. The GB is located at zero position. We evaluated the CL signals on the positive as well as on the negative side of the GB. According to ref. ^22^, the recombination velocity at GBs *S*_GB_ can be extracted by modeling the gradient of logarithm of the relative CL intensity Δ*I*.

6 $${\mathrm{ln}}\left[ {\Delta I(x)} \right] = {\rm{ln}}\left[ {S/(S + 1)} \right]-x/L,$$

where *L* is the electron diffusion length, *S* the reduced recombination velocity (*S* = *S*_GB_*τ*_GB_/*L*), and *x* the position of the electron beam. The extracted *S*_GB_ values were determined for a lifetime of 180 ns, as experimentally measured by the PL. For 20 different GBs in the Rb-containing CIGSe layer analyzed in the present work by CL, *S*_GB_ remained in the range of (0.02–5) × 10^3^ cm s^−1^ with an average value of about 500 cm s^−1^. We note that some *S*_GB_ values are on the same order of magnitude as the ones obtained on Rb-free/Na-containing or Rb-free/Na-free CIGSe layers (1000 cm s^−1^)^8,21^. Nevertheless, several GBs exhibit considerably smaller recombination velocities (10–100 cm s^−1^). Currently, we cannot conclude whether it is the carefully controlled CIGSe deposition process used for the CIGSe solar cell studied in the present work or rather Rb accumulation at the GBs during the RbF PDT that is responsible for the small, average *S*_GB_ value of 500 cm s^−1^.

On the other hand, the spreading of the values between 0.02 and 5 × 10^3^ cm s^−1^ can be explained easily by assuming that the recombination velocities at the GBs are effectively scaled by the band bending at the GB planes, exhibiting barriers for charge carriers with magnitude *Φ*_b_^23,24^:

7 $$S_{{\mathrm{GB,eff}}} = S_{{\mathrm{GB}}}{\mathrm{exp}}\left( { - {\it{\Phi}}_{\mathrm{b}}/k_{\mathrm{B}}T} \right),$$

where *S*_GB,eff_ is the effective GB recombination velocity (roughly determined by means of CL in the present work) and *S*_GB_ can be written as 1/2 *N*_GB_*σ*_e_*v*_th_ (which is the product of the density of defect states at the GB plane, the capture cross-section for electrons as minority charge carriers, and the thermal velocity). The barrier height *Φ*_b_ results from excess charge densities at the GB plane, which are either positive or negative. Free charge carriers (mainly holes in the *p*-type CIGSe layer) redistribute corresponding to the positive or negative nature of the GB charges, which leads via Poisson’s equation to a downward (negative) or upward (positive) bending of the conduction and valence bands. Since we can assume that, at different GBs, either positive or negative excess charge densities can be present with different density values, the corresponding barrier heights *Φ*_b_ also are broadly spread between positive and negative extrema, leading to the wide interval of *S*_GB,eff_ values (0.02 and 5 × 10^3^ cm s^−1^) centered around an average *S*_GB_ value of about 500 cm s^−1^ (where, according to Eq. (7), low *S*_GB,eff_ values are reached for positive barrier heights *Φ*_b_ and high *S*_GB,eff_ values for negative barrier heights *Φ*_b_). Upward band bending is more favorable for the device performance since it repels the electrons as minority-charge carriers from the GB plane; on the other hand, downward band bending drives free electrons toward the GB plane, leading to enhanced recombination and therefore to deteriorated solar cell performance.

In the two-dimensional device simulations, we use exactly this concept (Eq. (7)) to elucidate the influence of GBs on the device performance. For simplicity, we investigated this performance for *S*_GB_ = 100–500 cm s^−1^ and two effective barrier heights *Φ*_b_, +50 meV (upward band bending) and −50 meV (downward band bending).

### Absolute PL imaging and EQE

**Fig. 6:**
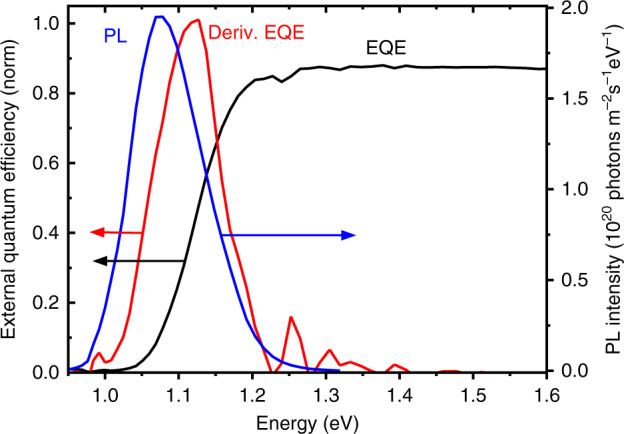
External quantum efficiency and photoluminescence. EQE spectrum (black) and the first derivative of the EQE curve (red) of the high-efficient solar cell as well as the average absolute PL spectrum (blue) acquired also on the identical solar cell at an excitation equivalent to the illumination intensity of one sun.

Figure 6 shows the average PL spectrum from the CIGSe solar cell integrated over the area of one individual cell, together with the EQE spectrum and its first derivative. For the PL spectrum, the emission is centered at 1.08 eV, coinciding with the minimum energy of the emission detected by the CL measurements on the cross-section of the device presented in the previous part (and about 30 meV lower than the band-gap energy determined from the EQE spectrum, see below). The average external PL quantum yield $Q_{\mathrm{e}}^{{\mathrm{PL}}}$ of the sample is about 1% under irradiation conditions equivalent to one sun. This PL quantum yield value predicts a nonradiative loss of *k*_B_*T* ln $\left( {Q_{\mathrm{e}}^{{\mathrm{PL}}}} \right)$ ≈ 120 meV^25,26^, which accounts for most of the difference between the *V*_oc_ = 720 mV measured for the CIGSe solar cell in the present work and the theoretical limit (see Table 1).

Following the procedure described by Redinger et al.^27^, we estimate the (effective) minority-carrier lifetime via

8 $$\tau _{\mathrm{n}} = 4n^2/\left( {B\,p_0} \right)\,\left( {Y_{{\mathrm{Laser}}}/Y_{{\mathrm{PL}}} - 1} \right)^{ - 1}.$$

The laser photon flux *Y*_Laser_ was about 2.5 × 10^21^ photons (m^2^ s eV)^−1^, and from absolute PL measurements, we obtained values for the integrated external PL photon flux *Y*_PL_ of about 2.5 × 10^19^ photons (m^2^ s eV)^−1^. Moreover, we assumed radiative recombination coefficients *B* of (0.6–1.2) × 10^−10^ cm^3^ s^−1^ (Equation (2.60) in ref. ^10^) and a refractive index *n* = 3.0^28^. By means of CV measurements in the section “Charge carrier collection behavior,” the net-doping density *p*_0_ in the CIGSe layer was determined to be about (2 ± 1) × 10^16^ cm^−3^. Altogether, we obtained minority-carrier lifetime values (*τ*_n_) of about 80–240 ns. Electron diffusion length values were determined to be about 3–8 µm, assuming diffusion constants of 1–3 cm^2^ s^−1^ (i.e., mobility values of 40–100 cm^2^ V s^−1^ ^10,16^).

The band-gap energy of the CIGSe layer can be determined from the peak energy of the first derivative of the EQE spectrum (Fig. 6), which was about 1.11 eV. The difference between this band-gap energy and the PL peak emission energy can be analyzed further. When calculating the PV band-gap energy of the CIGSe layer via the approach described by Rau et al.^29^, also 1.11 eV was obtained. This value agrees well also with the estimate of the minimum band-gap energy from the local minimum (notch) of the [Ga] distribution (see Fig. 2d). As outlined by Rau et al.^29^, it is possible to estimate also the amplitude *σ* of the band-gap fluctuations within the CIGSe layers from the first derivative of the EQE spectrum. The corresponding value for the EQE data shown in Fig. 6 was *σ* = 40 meV, which results (using Eq. (5)) in a *V*_oc_ loss of about 30 mV, regarded as radiative loss via band-gap broadening. These results give rise to the assumption that considerable band-gap fluctuations are indeed present within the CIGSe layer and that, since they were not detected at such a large amplitude *σ* by means of CL mapping, their length scale is on the order of a few 10 nm. Please note that adding the nonradiative *V*_oc_ loss of 120 mV and the one due to band-gap fluctuations of 30 mV yields good agreement between the measured *V*_oc_ and the theoretical value in Table 1.

For the following two-dimensional device simulations, we set the band-gap energy of CIGSe to 1.11 eV. Moreover, since radiative loss via band-gap broadening cannot be taken into account easily in the device simulation, it will be accounted for separately, as discussed in the following section “Two-dimensional device simulations”.

### Two-dimensional device simulations

For device simulations, the realistic microstructure depicted in Supplementary Fig. 3 was used. For the CdS buffer, the (Zn,Mg)O high resistive layer, and the ZnO:Al front contact, standard values were taken from Table 8.4 in ref. ^10^. The CIGSe/CdS interface was assumed to be mostly free of defects.

For the CIGSe absorber layer, the parameter values determined by the above described optoelectronic characterization were used, that is, the acceptor density, the band-gap profile, the GB recombination velocity, the charge carrier mobilities, and the charge carrier effective lifetimes. Values of other parameters, such as effective density of states, band offsets, or permittivity, were also taken from Table 8.4 in ref. ^10^. We summarized the material parameter values used for the CIGSe and additional layers in Supplementary Table 1. Variable simulation parameters are the intragrain minimum Shockley–Read–Hall lifetime, *τ*_bulk,nonrad_, accounting for nonradiative recombination of charge carriers via deep defects in the interior of the grains, the charge of GB defects (acceptor or donor), and the recombination velocity at the GB (see Eq. (7)).

The charge of GB defects has to be determined such that recombination of charge carriers at GB defects is consistent with the experimentally determined effective lifetime of 80–240 ns. To access the effective lifetime for a particular GB condition also in the simulation, we performed time-resolved PL (TRPL) simulations on the bare CIGSe absorber. Under low excitation conditions and in the absence of trap defects and electric fields, the time constant of the luminescence decay equals the effective minority charge-carrier lifetime^30–32^.

For a fixed (intragrain) bulk lifetime *τ*_bulk_ of 650 ns—i.e., the radiative limit, calculated by 1/*Bp*_0_—we varied the maximum GB recombination velocity and determined the time constant of the PL decay. We differentiated between acceptor-like defects 250 meV above the valence-band maximum (leading to a 50 meV upward band bending) and donor-like defects 250 meV below the conduction band minimum (leading to a 50 meV downward band bending). Supplementary Table 1 summarizes the simulated TRPL decay time for both cases as a function of the maximum GB recombination velocity.

By using the approximation *τ*_TRPL_ ≈ *τ*_eff_ and Matthiessen’s rule^33^

9 $$\frac{1}{{\tau _{{\mathrm{TRPL}}}}} \approx \frac{1}{{\tau _{{\mathrm{eff}}}}} = \frac{1}{{\tau _{{\mathrm{bulk}}}}} + \frac{1}{{\tau _{{\mathrm{GB}}}}}$$

with *τ*_bulk_ = 650 ns, we can calculate the lifetime *τ*_GB_ being assigned to GB recombination as a function of *S*_GB_. From Supplementary Table 1, we can infer that for *S*_GB_ = 500 cm s^−1^ and downward band bending, the theoretical lifetime *τ*_GB_ is smaller (21 ns) and that, for upward band bending, the simulated value is larger (620 ns) than the effective lifetime of 80–240 ns. Overall, for *S*_GB_ = 500 cm s^−1^, this interval of experimental values lies well within one of the theoretical values for *τ*_eff_ of about 20–320 ns. Realistically, GBs in CIGSe solar cells will never exhibit only upward or only downward band bending, since the resulting excess charges at GBs are either positive or negative. Therefore, a mixture of donor- and acceptor-like defects can be expected, leading to the measured effective lifetimes of 80–240 ns.

The next step is the determination of the intragrain carrier lifetime. This lifetime must be consistent with 110 ns resulting from recombination at GBs (see Supplementary Table 1 for *S*_GB_ = 500 cm s^−1^ and no band bending), an effective lifetime of 80–240 ns, and a *V*_oc_ of 750 mV, which is the experimentally measured value of 720 mV plus 30 mV estimated for the loss due to band-gap fluctuations.

The band bending at the GBs was set to 0 meV, and *τ*_bulk,nonrad_ as well as the effective GB recombination velocities were varied. From Supplementary Table 2, it can be seen that an effective lifetime *τ*_eff_ of 110–140 ns as well as recombination velocities of about 100–200 cm s^−1^ are required in order to realize *V*_oc_ values of about 750 mV (we used 200 cm s^−1^ for the following considerations). This theoretical interval of 110–140 ns is in good agreement with the experimental interval of 80–240 ns determined by PL. The short-circuit current densities in Supplementary Table 2 remain very similar to the experimental value of 36.6 mA cm^−2^ regardless of the parameter variations.

Knowing the effective lifetime (110 to 140 ns), the radiative lifetime of 650 ns, and the lifetime of 330 ns for GB recombination at *S*_GB_ = 200 cm s^−1^ (Supplementary Table 1), we used the extended Matthiessen’s rule

10 $$\frac{1}{{\tau _{{\mathrm{eff}}}}} = \frac{1}{{\tau _{{\mathrm{bulk}}}}} + \frac{1}{{\tau _{{\mathrm{GB}}}}} = \frac{1}{{\tau _{{\mathrm{bulk}},{\mathrm{rad}}}}} + \frac{1}{{\tau _{{\mathrm{bulk}},{\mathrm{nonrad}}}}} + \frac{1}{{\tau _{{\mathrm{GB}}}}}$$

and found that the intragrain, nonradiative lifetime *τ*_bulk,nonrad_ has to be around 200–500 ns for *τ*_eff_ = 110–140 ns.

We set *τ*_bulk,nonrad_ = 550 ns, *τ*_eff_ = 130 ns, and *S*_GB_ = 200 cm s^−1^ for the following calculations, i.e., it was assumed that recombination at GBs dominates the recombination processes in the CIGSe absorber layer. Supplementary Table 3 summarizes all derived simulation parameter. A simulation of the PV parameters using the model described above yields the values given in Table 1.

While the *j*_sc_ values match very well, the simulated *V*_oc_ and FF values as well as the conversion efficiency are too large as compared with the experiment. Good agreements are reached (values in the parentheses in Table 1) simply by taking into account the *V*_oc_ loss induced by the band-gap fluctuations (30 mV) and a consequently decreased FF, which was calculated using the formalism reported by Green^34^ as well as a diode-ideality factor of 1.4 and a series resistance of 0.5 Ω cm^2^ (determined from the *j*–*V* data of the investigated solar cell).

In order to study the behavior of GBs on the device performance, we used the reference model in Supplementary Table 3 and varied S_GB_ systematically from 0 to 5000 cm s^−1^ for upward band bending of +50 meV and downward band bending of −50 meV. The resulting PV parameters are summarized in Table 2.

**Table 2:** Simulated device performances depending on grain boundary recombination velocity.

*S*_GB_ (cm s^−1^)	*V*_oc_ (mV)	*j*_sc_ (mA cm^−2^)	FF (%)	*η* (%)
0 (and 0 meV band bending)	771	36.8	82	23.3
0	772–771	36.8	82	23.4–23.3
50	771–765	36.8–36.7	82	23.3–23.1
100	770–759	36.8–36.7	82	23.3–22.9
200	768–751	36.7	82	23.2–22.5
500	763–736	36.7–36.6	82–81	23.0–21.9
1000	756–721	36.7–36.5	82–81	22.8–21.2
2000	746–705	36.6–36.2	82–80	22.4–20.4
5000	729–684	36.5–35.6	82–79	21.7–19.1

Simulated solar cell parameters based on the material parameter values given in Supplementary Table 3 and using *S*_GB_ values ranging from 0 to 5000 cm s^−1^ as well as including upward band bending of +50 meV (upper limit) and downward band bending of −50 meV (lower limit of values).

The results in Table 2 show that, even for a high-efficient CIGSe solar cell, recombination at CIGSe GBs decreases the *V*_oc_ (and the FF) of the device substantially (and slightly also the *j*_sc_). For recombination velocities of 200–500 cm s^−^^1^, the *V*_oc_ values reside in the interval of 736–768 mV, which includes well the experimental value of 750 mV (720 + 30 mV, taking into account the radiative loss by the band-gap fluctuations). Indeed, this result is another strong indication that a mixture of upward as well as downward band bending is present at the various GBs in CIGSe thin films and that 50 meV is a good estimate for the average barrier height.

**Fig. 7:**
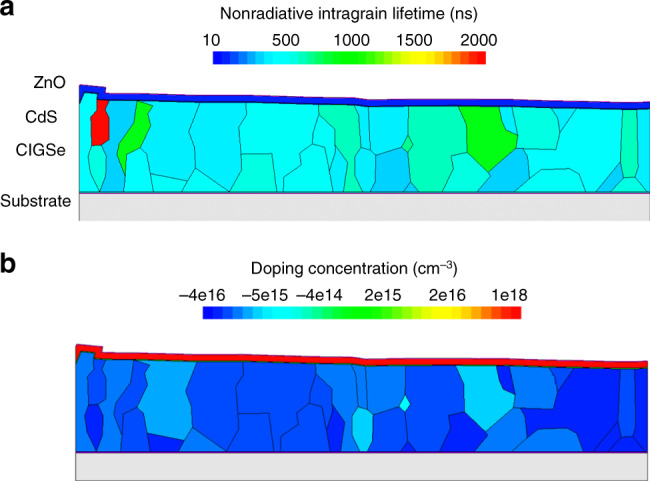
Device models including laterally varying lifetime and net-doping densities. **a** Distribution of electron lifetime and **b** doping concentration corresponding to the cross-sectional microstructure obtained by EBSD (Supplementary Fig. 3). Simulation is based on Gaussian distributed experimental values. Note that negative doping concentrations denote those of acceptors, whereas positive values are those of donors.

The effect of laterally varying electron lifetime and net-doping concentration as inhomogeneities in the CIGSe absorber were studied using *τ*_bulk,nonrad_ = 550 ns and *S*_GB_ = 200 cm s^−1^. We would like to note that we studied this effect also on absorbers with *S*_GB_ = 0 cm s^−1^; however, we did not find any difference to the model applied in the present subsection with respect to the impact of fluctuating electron lifetime and net-doping concentrations on the device performance. A random number generator was used, which distributed the nonradiative intragrain lifetime and/or the net-doping density randomly and independently to each grain in the simulated microstructure (Supplementary Fig. 3b). In the present work, we use Gaussian distributions with (550 ± 500) ns for *τ*_bulk,nonrad_ (corresponding to an error of 60 ns for the effective lifetime) and (2 ± 1) × 10^16^ cm^−3^ for the net-doping density (according to the result in the section “Charge carrier collection behavior”). Here we use the 60-ns variance of the lifetime (from PL results) as the standard deviation for the lifetime fluctuation. The resulting electron-lifetime and net-doping distribution maps are shown in Fig. 7.

Using the models depicted in Fig. 7, we computed illuminated *j*–*V* curves and compared them with *j*–*V* curves calculated for CIGSe absorbers without inhomogeneities. Table 3 summarizes the PV parameters for the four different cases.

**Table 3:** Simulated photovoltaic parameters of cells with various inhomogeneities.

Inhomogeneities	*V*_oc_ (mV)	*j*_sc_ (mA cm^−2^)	FF (%)	*η* (%)
Without	750	36.7	82	22.4
Only doping density	748	36.7	82	22.3
Only electron lifetime	749	36.7	82	22.4
Both	747	36.7	82	22.3

Solar cell parameters extracted from simulated, illuminated *j*–*V* curves with respect to the approximated grain structure. Simulation results are divided into different aspects of lateral inhomogeneities.

From Table 3, it is apparent that the PV parameters are hardly affected by the fluctuations in electron lifetime or in net-doping density. Therefore, these inhomogeneities are considered irrelevant for the device performance of high-efficient CIGSe solar cells.

## Discussion

The two-dimensional device simulation revealed that lateral inhomogeneities in lifetime and net-doping have no significant impact on the device performance of the highly efficient CIGSe solar cell studied in the present work. Nevertheless, when evaluating electrostatic potential fluctuations from the EBIC data in Fig. 2c, it was shown that these fluctuations result in a slight decrease of the *V*_oc_. Indeed, the contributions of the donor densities on the *n*-type side of the *p*–*n* junction, *N*_D,W_, and also the density of interface charges at the *p*–*n* junction, *N*_IF_, on the electrostatic potential fluctuations are not negligible; since the simulation indicates that fluctuations in *N*_A,a_ (the net-doping density in the CIGSe absorber) do not affect the PV parameters considerably, we can conclude that the main contribution to electrostatic potential fluctuations can be attributed to lateral variations in *N*_D,w_ and *N*_IF_.

As opposed to the lateral inhomogeneities in lifetime and net-doping, band-gap fluctuations on the order of 40–45 meV were found to affect the *V*_oc_ substantially via radiative losses (as already proposed by Rau et al.^13^), which amount to a decrease of 30 mV for the studied high-efficiency solar cell. We note that the assumed length scale of these fluctuations of few 10 nm makes it very difficult to detect them via microscopic luminescence techniques. Nevertheless, it is possible to estimate the corresponding losses by evaluating EQE spectra of the CIGSe solar cells by the procedure described in ref. ^25^. Moreover, band-gap energies of CIGSe absorbers (i.e., the minimum band-gap energy at the notch position) can be determined reliably also from the EQE data, as already reported by Carron et al.^35^, in contrast to from PL spectra.

GBs have a significant impact on the device performance. When compared with the average grain size in the CIGSe layer of 0.5 µm (EBSD measurements), *L*_e_ is at least one order of magnitude larger (revealed by PL spectroscopy). This result agrees well with the fact that high *j*_sc_ values are possible in spite of the small grain size. Still, the *V*_oc_ and the FF values are decreased substantially (as compared with a simulated, GB-free device) by enhanced nonradiative recombination at GBs^36^, although the Rb-containing CIGSe solar cell studied in the present work exhibits low average *S*_GB_ values (200–500 cm s^−1^). Currently, we do not have a conclusive explanation for these small average *S*_GB_ values. Whether they can be traced back to the Rb accumulation at GBs after the RbF PDT^37^, or rather to the carefully controlled CIGSe growth process, will be the object of further investigations. For a complete picture of the enhanced nonradiative recombination at GBs and its possible passivation, it is important to investigate the impact of all the numerous point defects accumulating at the GBs for various CIGSe layers with different chemical compositions, and not only of those point defects that are related to individual impurity elements.

In this respect, we would like to note that Siebentritt et al.^38^ reported evidence for the passivating effect of K, Rb, and Cs at GBs (via the reduction of barriers *Φ*_b_ for charge carriers), while Abou-Ras et al.^39^, using CL analyses, did not confirm that a KF PDT of CIGSe surfaces leads to passivation of the GBs in these absorber layers (i.e., the effective recombination velocities *S*_GB,eff_ remain at about the same level with and without the KF PDT). This discrepancy can be traced back at least in part to the fact that Siebentritt et al. applied surface-sensitive scanning Kelvin-probe force microscopy for the measurement of the barriers heights *Φ*_b_ at the GBs and that GB properties at the very surface of a CIGSe layer need not be the same as in the CIGSe bulk. Moreover, also the secondary phases forming on top of the CIGSe layers upon KF, RbF, or CsF PDT can be expected to influence the GB properties at the CIGSe surface substantially.

On the other hand side, both the experimental (CL) as well as the simulation results in the present work are consistent with the assumption that the GBs in the CIGSe thin film within the investigated highly efficient solar cell exhibit recombination velocities of few 100 cm s^−1^ in average and a mixture of upwards and downwards bended energy bands, for which the absolute values of the barrier heights are about 50 meV. The good agreement of the two-dimensional device simulations with the experimental values (except for the FF), given in Table 2, are obvious, especially when considering that the “real” PV parameters reside somewhere between the two extremes of only upward and only downward band bending and also that the value of *S*_GB,eff_ = 200 to 500 cm s^−1^ (and also other simulation parameters such as the electron lifetime) is not precise but a rough estimation.

It should be noted that the experimental values in the present work were simulated successfully assuming the nonradiative intragrain lifetime *τ*_bulk,nonrad_, 500 ns, to be close to the radiative intragrain lifetime *τ*_bulk,rad_ of 650 ns. This is, nonradiative recombination at GBs (with lifetime of about 300 ns) can be considered the dominant contribution to the effective electron lifetime of about 110–140 ns.

In conclusion, the present work reported about the combination of various and complementary characterization techniques applied to a high-efficient CIGSe solar cell (about 21% without ARC), which comprises a Rb-containing CIGSe absorber layer. Corresponding results were used as input parameters for two-dimensional device simulations, which were based on a real microstructure of the polycrystalline CIGSe layer as provided by an EBSD map. The CIGSe absorber exhibited effective electron lifetimes of about 100 ns (order of magnitude) and a high $Q_{\rm{e}}^{{\mathrm{PL}}}$ value of 1%.

It is shown that lateral inhomogeneities in net-doping density and electron lifetime have no substantial impact on the device performance. In contrast, band-gap fluctuations of 40–45 meV decrease the *V*_oc_ by about 30 mV. Moreover, enhanced recombination at GBs decrease the *V*_oc_ and FF of the device (compared with their theoretical limits) substantially, although rather low, average (effective) recombination velocities at GBs of several 100 cm s^−1^ are determined (as compared with values of several 1000 cm s^−1^ measured on Na/K-free or Na/K-containing CIGSe layers without any subject to RbF PDT^39^. It will be matter of future studies to reveal whether these low *S*_GB_ values can indeed be traced to a passivation effect of Rb at CIGSe GBs. The approach applied in the present work may be transferred to similar studies on other optoelectronic semiconductor devices.

## Methods

### Solar cell fabrication

For the production of high-efficiency CIGSe solar cells, we use aluminosilicate glass substrates that are cut and washed before deposition of a thin film of sputtered Mo (500–900 nm). An important step was the coevaporation of Cu, In, Ga, and Se to grow the CIGSe semiconductor layer in a multi-stage process (2.5–3.0 μm). Subsequently, we performed an alkali PDT with RbF. Then the samples were taken out of the vacuum chamber in order to coat the CIGSe with a thin layer of CdS (25–40 nm) in a chemical bath. We sputtered (Zn,Mg)O (50–100 nm) and Al-doped ZnO (150–200 nm) window layers. Finally, a Ni/Al/Ni grid for contacting the completed cell was evaporated. The resulting cell area was about 0.5 cm^2^.

### SEM analyses

For microscopic analyses, specimens were prepared by cutting slices of a few millimeters in width from the ZnO:Al/(Zn,Mg)O/CdS/CIGSe/Mo/glass stack, gluing each two of these slices together face to face, and polishing the cross-sections mechanically. In order to reduce the sample drift during data acquisition, very thin carbon layers (nominally 5 nm) were deposited on the cross-section surface.

The characterization by means of EBSD, EDX, EBIC, and CL was carried out using Zeiss UltraPlus and Zeiss Merlin scanning electron microscopes. These instruments were equipped with Oxford Instruments NordlysNano and XMax80 detectors, a point electronic EBIC amplifier including a lock-in amplifier, and a DELMIC SPARC CL system. The EBSD measurements were performed using a beam energy of 20 keV. EBSD patterns were acquired at point-to-point distances of 50 nm and evaluated using the Oxford Instruments/HKL software packages AZtec and CHANNEL5. Note that, since the used EBSD software was not able to index the tetragonal crystal structure of CIGSe correctly^40^, a pseudocubic symmetry was used for the indexing of the EBSD patterns. Elemental distribution maps via EDX were acquired at 7 keV and evaluated by the AZtec software suite (Oxford Instruments).

The EBIC signals were recorded by the acquisition and evaluation software DIPS5 (point electronic). Moreover, a beam blanker at a frequency of 5 kHz was used to increase the signal-to-noise ratio. In order to avoid high-injection conditions, EBIC signals were acquired at a beam current of only a few tens of pA; also, the EBIC measurements were carried out at short durations (<30 min) to impede effects of long illumination by electron beam, such as electron-beam soaking^41–43^. The beam energies were varied between 6 and 15 keV to estimate the influence of the surface recombination. During the course of the present work, we did not detect any EBIC kinks or similar artifacts^42^. The CL measurements were performed at room temperature using a beam current of 1 nA and a beam energy of 10 keV. All measurements were performed at room temperature (about 25 °C), similar to standard solar cell test conditions.

As for the injection conditions during EBIC and CL analyses, we can estimate them roughly using the equation proposed by Maurice and Marfaing^44^ and outlined in a recent EBIC tutorial by Abou-Ras and Kirchartz^9^. For the calculations, we assume an electron lifetime of 140 ns and a minimum band-gap energy in the CIGSe absorber of 1.11 eV. The corresponding injection levels are as follows. For EBIC, the density of charge carriers by the electron beam were about 1 × 10^16^ cm^−3^ for a beam energy of 6 keV and about 1 × 10^15^ cm^−^^3^ for 12–15 keV. For CL, since a higher beam current of 500–1000 pA was applied to achieve a decent signal-to-noise ratio, even at 10 keV, the density of generated electron–hole pairs reaches values of about (2–5) × 10^16^ cm^−3^. Although for the EBIC measurements at low energies (6 keV) and for the CL analyses the generated densities of electron–hole pairs are on the same order of magnitude as the net-doping concentrations, no clear high-injection conditions were present, for which even higher densities of electron–hole pairs need to be generated by the incident electron beam.

### PL and capacitance measurements

For the PL imaging measurements, the Mo/CIGS/CdS/(Zn,Mg)O/ZnO:Al samples were excited optically using red light from two 660-nm lasers coupled to homogenizer units. The excitation beam was adjusted to be equivalent to 1-sun conditions for an assumed, step-like absorptivity corresponding to a band-gap energy of about 1.1 eV (2.5 × 10^21^ photons m^−2^ s^−1^ eV^−1^). Spectral resolution of the PL was obtained using a liquid crystal, tunable filter with a wavelength step size of 10 nm. The PL images from 900 to 1700 nm were taken via a peltier-cooled InGaAs camera calibrated to detect the absolute photon numbers. CV measurements were performed at room temperature using a HP4284 LCR-Meter and frequencies of 1 MHz.

### Two-dimensional device modeling

The precise modeling of a polycrystalline CIGSe solar cell requires a real CIGSe grain structure. This grain structure can be accessed from EBSD. Supplementary Fig. 3 illustrates how a simulation model is obtained from such a microscopic image of a CIGSe cross-section.

First, we started with a cross-section of the CIGSe absorber and then the grains were approximated by polygons using an image processing software. With the aid of a digitizer, the grain coordinates were stored in a list, which was inserted into the simulation tool Sentaurus TCAD. Thus the absorber was defined, and a buffer layer and a window layer were deposited conformally onto it. After definition of the geometry, material properties were assigned to each region. Hence, each CIGSe grain may have a different band-gap energy, lifetime, doping density, or may even consist of a totally different material. By this approach, we were able to mimic inhomogeneities and secondary phases on the submicrometer scale. Eventually, the optical generation function was computed via raytracing, a mathematical mesh was created, and the semiconductor equations, i.e., Poisson’s equation and the two continuity equations, were discretized and solved numerically.

### Reporting summary

Further information on research design is available in the Nature Research Reporting Summary linked to this article.

## Supplementary information

Supplementary Information

Reporting Summary

## Data Availability

The data that support the findings of this study are available from the corresponding author upon reasonable request.
