# Author Correction: Microscopic origins of performance losses in highly efficient Cu(In,Ga)Se_2_ thin-film solar cells

**DOI:** 10.1038/s41467-021-23652-5

**Published:** 2021-05-21

**Authors:** Maximilian Krause, Aleksandra Nikolaeva, Matthias Maiberg, Philip Jackson, Dimitrios Hariskos, Wolfram Witte, José A. Márquez, Sergej Levcenko, Thomas Unold, Roland Scheer, Daniel Abou-Ras

**Affiliations:** 1 Helmholtz-Zentrum Berlin, Hahn-Meitner-Platz 1, 14109 Berlin, Germany; 2 Institute of Physics, Martin-Luther University Halle-Wittenberg, Von-Danckelmann-Platz 3, 06120 Halle, Germany; 3 Zentrum für Sonnenenergie- und Wasserstoff-Forschung Baden-Württemberg (ZSW), Meitnerstr. 1, 70563 Stuttgart, Germany

**Keywords:** Solar cells, Solar cells, Characterization and analytical techniques

Correction to: *Nature Communications* 10.1038/s41467-020-17507-8, published online 21 August 2020.

The original version of this Article contained errors in Table 1. The correct version of the 4th row of the 1st column states ‘720 (750)’ instead of the original, incorrect ‘750 (720)’; the 4th row of the 3rd column states ‘80 (82)’ instead of the original, incorrect ‘82 (80)’ and the 4th row of the 4th column states ‘21.1 (22.4)’ instead of the original, incorrect ‘22.4 (21.1)’.

The correct version of the fourth sentence in the caption of Table 1 states “The *V*_oc_ value in parentheses (720 mV) is the one for a CIGSe layer exhibiting band-gap fluctuations.” instead of the original, incorrect “The *V*_oc_ value in parentheses is the one for a CIGSe layer free of band-gap fluctuations.”.

The first sentence of the sixth paragraph of the subsection “Cathodoluminescence (CL) behavior of the CIGSe absorber” in Results contained an error and originally incorrectly read “*S*_GB_ can be written as 1/4*N*_GB_*σ*_e_*v*_th_”. The correct version reads “*S*_GB_ can be written as 1/2*N*_GB_*σ*_e_*v*_th_”.

This has been corrected in both the PDF and HTML versions of the Article.

