# Supplementary material for: Microscopic origins of performance losses in highly efficient Cu(In,Ga)Se2 thin-film solar cells
Source: Nat Commun. 2020 Aug 21;11:4189. doi: 10.1038/s41467-020-17507-8 (PMC7442832; doi:10.1038/s41467-020-17507-8)
Supplement: Supplementary file 1 — Supplementary Information [file 41467_2020_17507_MOESM1_ESM.pdf]

Supplementary Information

Microscopic origins of performance losses in highly efficient $Cu(In,Ga)Se_2$ thin-film solar cells

Maximilian Krause[1], Aleksandra Nikolaeva[1], Matthias Maiberg[3], Philip Jackson[2], Dimitrios Hariskos[2], Wolfram Witte[2], José A. Márquez[1], Sergej Levcenco[1], Thomas Unold[1], Roland Scheer[3], Daniel Abou-Ras[1*]

[1] Helmholtz-Zentrum Berlin, Hahn-Meitner-Platz 1, 14109 Berlin, Germany

[2] Zentrum für Sonnenenergie- und Wasserstoff-Forschung Baden-Württemberg (ZSW), Meitnerstr. 1, 70563 Stuttgart, Germany

[3] Institute of Physics, Martin-Luther University Halle-Wittenberg, Von-Danckelmann-Platz 3, 06120 Halle, Germany

*Corresponding author: daniel.abou-ras@helmholtz-berlin.de

1. Supplementary Figures

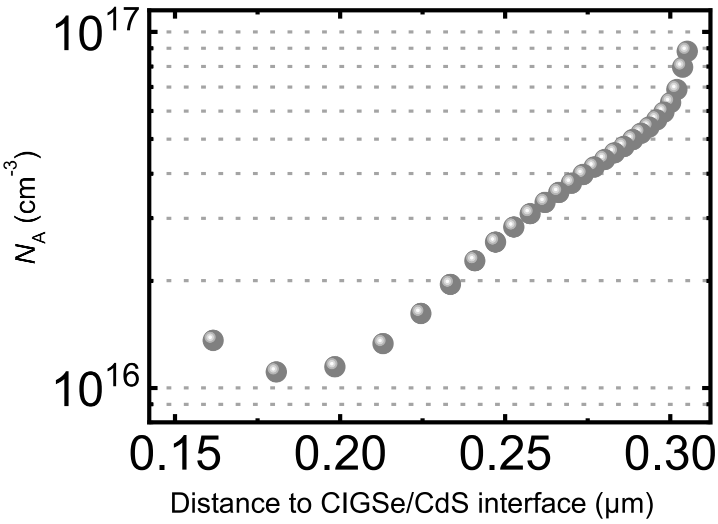

Supplementary Figure 1: **Net-doping density in the CIGSe layer.** Charge carrier concentration N_A in the analyzed CIGSe thin film as a function of the distance to the CIGSe/CdS interface.

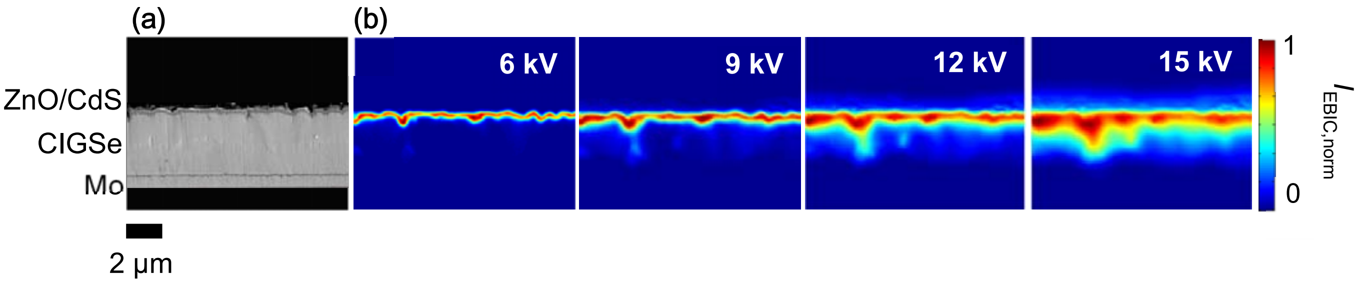

Supplementary Figure 2: **Charge-carrier collection in dependence of acceleration voltage.** Cross-sectional SEM image (a) and corresponding (b) EBIC signals recorded at various acceleration voltages (6, 9, 12, and 15 kV).

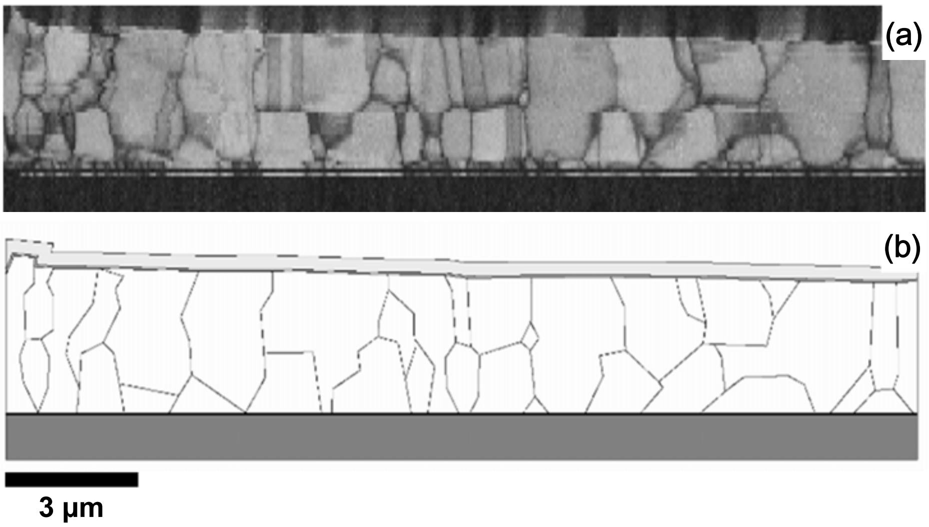

Supplementary Figure 3: **Microstructure used for simulation.** (a) EBSD quality-pattern map obtained from cross-section of a CIGSe absorber and (b) digitization of the polygon corners for the input into Sentaurus TCAD.

2. Supplementary Tables

Supplementary Table 1: **Simulated lifetimes.** Simulated lifetimes τ_{TRPL} of time-resolved photoluminescence on a blank absorber as a function of the maximum GB recombination velocity S_{GB}. Three cases of 50 meV upward and downward band bending, realized by acceptor-like and donor-like defects, as well as zero band bending realized by neutral defects were distinguished. By application of Equation (9) and using τ_{bulk} = 650 ns, the lifetime τ_{GB}, which is related to GB recombination, was determined.

S_{GB} (cm s^{-1})	50 meV upward band bending		No band bending		50 meV downward band bending	
	τ_{TRPL} (ns)	τ_{GB} (ns)	τ_{TRPL} (ns)	τ_{GB} (ns)	τ_{TRPL} (ns)	τ_{GB} (ns)
50	555	3800	399	1030	141	180
200	470	1700	220	330	54	59
500	317	620	93	110	20	21
2000	158	210	36	38	8	8
5000	64	71	16	16	4	4

Supplementary Table 2: **Impact of various nonradiative time-constants.** Simulation of the V_{oc} and the j_{sc} values for a solar cell containing a CIGSe absorber with S_{GB} values of 100, 200, and 500 cm s^{-1} and lifetimes $\tau_{bulk,nonrad}$ for nonradiative intragrain recombination ranging from 50 to 500 ns. The τ_{eff} columns were calculated using Equation (9), with $\tau_{bulk,rad}$= 650 ns and τ_{GB} = 490 ns (100 cm s^{-1}), 250 ns (200 cm s^{-1}), 110 ns (500 cm s^{-1}).

$\tau_{bulk,nonrad}$ (ns)	100 cm s^{-1}			200 cm s^{-1}			500 cm s^{-1}		
	τ_{eff} (ns)	V_{oc} (mV)	j_{sc} (mA cm^{-2})	τ_{eff} (ns)	V_{oc} (mV)	j_{sc} (mA cm^{-2})	τ_{eff} (ns)	V_{oc} (mV)	j_{sc} (mA cm^{-2})
50	42	717	36.4	39	715	36.4	33	709	36.3
100	74	732	36.6	64	729	36.5	48	720	36.5
200	120	745	36.7	95	740	36.6	64	728	36.5
300	140	751	36.7	110	745	36.7	72	731	36.6
500	180	757	36.7	130	749	36.7	79	733	36.6
1000	220	763	36.7	150	753	36.7	86	736	36.6

Supplementary Table 3: **Simulation parameters.** The most relevant CdS buffer and CIGSe absorber materials-parameter values used for the two-dimensional simulations.

Simulation parameter	Values
$E_{g,ZnO}$	3.4 eV
$E_{g,CdS}$	2.4 eV
$E_{g,CIGSe}$ (minimum)	1.11 eV
Average conduction band offset at absorber/buffer	-0.1 eV
conduction band offset at buffer/window	0.1 eV
Acceptor density, $N_{A,a}$	$2x10^{16}$ cm^{-3}
Charge carrier mobility, μ	40 cm^2 Vs^{-1}
Coefficient for radiative recombination, B	$7x10^{-11}$ cm^3 s^{-1}
Intragrain, non-radiative recombination, $\tau_{bulk,nonrad}$	500 ns
Gap states at GBs, N_{GB}	10^9 cm^{-2} (neutral) at mid-gap
GB recombination velocity, S_{GB}	200 cm s^{-1}
Back-contact recombination for electrons and holes, $S_{b,n}$, $S_{b,p}$	0.1 cm s^{-1}, 10^7 cm s^{-1}
Front-contact recombination for electrons and holes, $S_{f,n}$, $S_{f,p}$	10^7 cm s^{-1}
Recombination at all other interfaces, S_{IF}	0.1 cm s^{-1}
Series resistance	0.5 Ωcm^2
