# Supplementary material for: Microscopic origins of performance losses in highly efficient Cu(In,Ga)Se2 thin-film solar cells
Source: Nat Commun. 2020 Aug 21;11:4189. doi: 10.1038/s41467-020-17507-8 (PMC7442832; doi:10.1038/s41467-020-17507-8)
Supplement: Supplementary file 2 — Reporting Summary [file 41467_2020_17507_MOESM2_ESM.pdf]

nature research

Corresponding author(s): Daniel Abou-Ras

Solar Cells Reporting Summary

Nature Research wishes to improve the reproducibility of the work that we publish. This form is intended for publication with all accepted papers reporting the characterization of photovoltaic devices and provides structure for consistency and transparency in reporting. Some list items might not apply to an individual manuscript, but all fields must be completed for clarity.

For further information on Nature Research policies, including our data availability policy, see Authors & Referees.

ü Experimental design

Please check: are the following details reported in the manuscript?

1. Dimensions

Item	Yes	No	Details
Area of the tested solar cells	☒	☐	Methods
Method used to determine the device area	☐	☒	Not relevant for the conclusions made in the present work.

2. Current-voltage characterization

Item	Yes	No	Details
Current density-voltage (J-V) plots in both forward and backward direction	☐	☒	Not relevant for the conclusions made in the present work.
Voltage scan conditions *For instance: scan direction, speed, dwell times*	☐	☒	Not relevant for the conclusions made in the present work.
Test environment *For instance: characterization temperature, in air or in glove box*	☒	☐	Methods
Protocol for preconditioning of the device before its characterization	☐	☒	Not relevant for the conclusions made in the present work.
Stability of the J-V characteristic *Verified with time evolution of the maximum power point or with the photocurrent at maximum power point; see ref. 7 for details.*	☐	☒	Not relevant for the conclusions made in the present work.

3. Hysteresis or any other unusual behaviour

Item	Yes	No	Details
Description of the unusual behaviour observed during the characterization	☐	☒	Not relevant for the conclusions made in the present work.
Related experimental data	☐	☒	Not relevant for the conclusions made in the present work.

4. Efficiency

Item	Yes	No	Details
External quantum efficiency (EQE) or incident photons to current efficiency (IPCE)	☒	☐	Sec. 2.6, Figure 7
A comparison between the integrated response under the standard reference spectrum and the response measure under the simulator	☐	☒	Not relevant for the conclusions made in the present work.
For tandem solar cells, the bias illumination and bias voltage used for each subcell	☐	☒	No tandem solar cells reported

5. Calibration

Item	Yes	No	Details
Light source and reference cell or sensor used for the characterization	☒	☐	Not relevant for the conclusions made in the present work.
Confirmation that the reference cell was calibrated and certified	☐	☒	Not relevant for the conclusions made in the present work.

Calculation of spectral mismatch between the reference cell and the devices under test

- [ ] Yes
- [x] No

Not relevant for the conclusions made in the present work.

6. Mask/aperture

Size of the mask/aperture used during testing

- [ ] Yes
- [x] No

Not relevant for the conclusions made in the present work.

Variation of the measured short-circuit current density with the mask/aperture area

- [ ] Yes
- [x] No

Not relevant for the conclusions made in the present work.

7. Performance certification

Identity of the independent certification laboratory that confirmed the photovoltaic performance

- [ ] Yes
- [x] No

Not relevant for the conclusions made in the present work.

A copy of any certificate(s)
Provide in Supplementary Information

- [ ] Yes
- [x] No

Not relevant for the conclusions made in the present work.

8. Statistics

Number of solar cells tested

- [ ] Yes
- [x] No

Only one solar cell investigated in the present work.

Statistical analysis of the device performance

- [ ] Yes
- [x] No

Only one solar cell investigated in the present work.

9. Long-term stability analysis

Type of analysis, bias conditions and environmental conditions
For instance: illumination type, temperature, atmosphere humidity, encapsulation method, preconditioning temperature

- [ ] Yes
- [x] No

Not relevant for the conclusions made in the present work.
